# Astragaloside IV restrains pyroptosis and fibrotic development of pulmonary artery smooth muscle cells to ameliorate pulmonary artery hypertension through the PHD2/HIF1α signaling pathway

**DOI:** 10.1186/s12890-023-02660-9

**Published:** 2023-10-12

**Authors:** Jie Xi, Yan Ma, Dongmei Liu, Rong Li

**Affiliations:** 1 Outpatient department, Urumqi Youai Hospital, Xinjiang Uygur Autonomous Region, Urumqi, 830063 China; 2 Department of Critical Care Medicine, Urumqi Youai Hospital, Urumqi, 830063 Xinjiang Uygur Autonomous Region China; 3 Department of Gynaecology, Urumqi Maternal and Child Health Care Hospital, Xinjiang Uygur Autonomous Region, Urumqi, 830063 China; 4 Traditional Chinese Medicine department, Urumqi Maternal and Child Health Care Hospital, Xinjiang Uygur Autonomous Region, Urumqi, 830063 China; 5 Department of Critical Care Medicine, Urumqi Youai Hospital, Xinjiang Uygur Autonomous Region, No. 3838, Convention and Exhibition Avenue, Midong District, Urumqi, 830063 China

**Keywords:** Astragaloside, Pulmonary artery hypertension, Prolyl-4-hydroxylase 2, Hypoxia inducible factor-1α, Pyroptosis

## Abstract

**Background:**

Astragaloside (AS)-IV, extracted from traditional Chinese medicine *Astragalus mongholicus*, has been widely used in the anti-inflammatory treatment for cardiovascular disease. However, the mechanism by which AS-IV affects pulmonary artery hypertension (PAH) development remains largely unknown.

**Methods:**

Monocrotaline (MCT)-induced PAH model rats were administered with AS-IV, and hematoxylin-eosin staining and Masson staining were performed to evaluate the histological change in pulmonary tissues of rats. Pulmonary artery smooth muscle cells (PASMCs) were treated by hypoxia and AS-IV. Pyroptosis and fibrosis were assessed by immunofluorescence, western blot and enzyme-linked immunosorbent assay.

**Results:**

AS-IV treatment alleviated pulmonary artery structural remodeling and pulmonary hypertension progression induced by MCT in rats. AS-IV suppressed the expression of pyroptosis-related markers, the release of pro-inflammatory cytokine interleukin (IL)-1β and IL-18 and fibrosis development in pulmonary tissues of PAH rats and in hypoxic PAMSCs. Interestingly, the expression of prolyl-4-hydroxylase 2 (PHD2) was restored by AS-IV administration in PAH model in vivo and in vitro, while hypoxia inducible factor 1α (HIF1α) was restrained by AS-IV. Mechanistically, silencing PHD2 reversed the inhibitory effect of AS-IV on pyroptosis, fibrosis trend and pyroptotic necrosis in hypoxia-cultured PASMCs, while the HIF1α inhibitor could prevent these PAH-like phenomena.

**Conclusion:**

Collectively, AS-IV elevates PHD2 expression to alleviate pyroptosis and fibrosis development during PAH through downregulating HIF1α. These findings may provide a better understanding of AS-IV preventing PAH, and the PHD2/HIF1α axis may be a potential anti-pyroptosis target during PAH.

**Supplementary Information:**

The online version contains supplementary material available at 10.1186/s12890-023-02660-9.

## Introduction

Pulmonary artery hypertension (PAH) is a multifactorial pulmonary vascular disease with abnormally high pulmonary arterial pressure [1]. It is characterized by progressive pulmonary artery remodeling, resulting in increased right ventricular pressure overload, and even right ventricular failure and death [2, 3]. Although drugs such as endothelin receptor blockers and prostacyclin have improved the prognosis of patients with PAH, the 3-year mortality of patients with PAH is still up to 20–30% [4, 5]. There is an urgent need for the development of innovative drugs with potential therapeutic value to effectively improve the clinical treatment effect of PAH.

Traditional Chinese medicine has the advantages of safe, efficient and low-cost in the treatment of chronic diseases. It has potential development value to explore drugs for PAH from traditional Chinese medicine. Astragaloside (AS), a traditional Chinese medicine, has been used to treat cardiovascular diseases for more than 2000 years [6, 7]. AS-IV is the most active triterpenoid glycoside of *Astragalus membranaceus*, and has a variety of pharmacological effects, including anti-oxidation, anti-atherosclerosis, anti-cancer and enhancing immune function, etc. [8]. Recent research found that AS-IV has protective effect on PAH, such as attenuating hypoxia‑induced inflammation and pulmonary vascular remodeling [9, 10]. However, the protective mechanism of AS-IV in PAH has not been fully understood.

Chronic pulmonary inflammatory response is an important pathogenic mechanism leading to pulmonary artery remodeling and the progression of PAH [11, 12]. Immune cells and cytokines were found to infiltrate around the blood vessels of PAH patients and animal models [13, 14]. Inflammasome is multi-protein complexes that regulates cytokine maturation, inflammation and death, which plays a key role in innate immunity [15]. Nucleotide-binding oligomerization segment-like receptor family 3 (NLRP3) is the most characteristic inflammasome. Its activation will cleave caspase-1, thereby promoting downstream cytokines (such as pro-IL-1β and pro-IL-18) to be cleaved into their bioactive forms and released into the extracellular space through the plasma membrane pores [16, 17]. Pyroptosis, as an inflammatory cell necrosis, is mediated by NLRP3 inflammasome activation. Accumulating evidence indicates that pyroptosis is responsible for plenty of diseases progresses, such as nervous system related diseases and cardiovascular diseases [18–21]. In recent years, a few researches have reported that pyroptosis might contribute to pulmonary hypertension [11, 22–24]. In addition, NLRP3 inflammasome activation and its subsequent pyroptosis can also cause pulmonary fibrosis, and they drive pulmonary artery remodeling together [1]. Previous studies have pointed out that AS-IV could suppress NLRP3 inflammasome activation induced by high glucose and ischemia [25, 26], but its influence in PAH is rarely reported.

Hypoxia is the key pathogenic mechanism leading to pulmonary artery remodeling during PAH progression. Hypoxia inducible factor 1α (HIF1α), as a critical molecular signal for mammalian cells to response to hypoxia, can increase oxygen transport or promote metabolism to adapt to hypoxia by activating the transcription of various downstream genes involved in metabolism, angiogenesis, apoptosis, and other [27, 28]. Therefore, HIF1α plays a pivotal role in the pathophysiology of ischemic diseases. HIF1α is reported to be overactivated in hypoxia-induced PAH that can lead to changes in the phenotype of lung cells and remodeling of lung structure [29, 30]. Prolyl-4-hydroxylase 2 (PHD2) is one of the most important isoenzymes under normoxic conditions responsible for HIF1α hydroxylation and degradation. Recent evidence suggests deficiency of PHD2 induces pulmonary vascular remodeling and PAH progression [31–33]. In this research, we revealed that AS-IV elevated the expression of PHD2 to alleviate PAH progression through inhibiting HIF1α and NLRP3 inflammasome, which was not clarified in the previous publications. The present article clearly clarifies the mechanism by which AS-IV prevents PAH progression through pyroptosis pathway, and it might provide a theoretical basis for the development of effective prevention and treatment strategies of PAH.

## Materials and methods

### Animal models

Eighteen Sprague-Dawley rats with specific pathogen free (Male, 300 g ~ 350 g), purchased from Saiye model Biology Research Center Co., Ltd (Taicang, Jiangsu, LIC. SCXK(Su)2018-0003), were kept in cages with free access to water and food. The rats were divided into 3 groups (6 rats in each group): negative control (NC) group, PAH group and AS group. 60 mg/kg of monocrotaline (MCT, MedChemExpress, New Jersey, USA, HY-N0750) was injected intraperitoneally into rats in PAH group and AS group for the establishment of PAH models, and same volume of saline was injected into rats in NC group. The model was established successfully when the mean pulmonary artery pressure (mPAP) was greater than 25 mmHg. 24 h after MCT administration, 30 mg/kg of AS-IV (Yuanye, Shanghai, China, #B20564, dissolved in 0.5% DMSO) or the same volume of 0.5% DMSO in saline was administered intraperitoneally into rats for 4 w. Rats were anesthetized by intraperitoneal injection of tribromoethanol (300 mg/kg), mPAP and the weight ratio of the right ventricle to the left ventricle plus the septum [RV/(LV + S)] was measured as previous described [34].

### Hematoxylin-eosin staining

Rat lung artery tissue were firstly made into paraffin embedded tissue section, and then hematoxylin-eosin staining was performed as previous described [35]. The section was dewaxed, hydrated and stained by hematoxylin (Solarbio, Beijing, China, #G1120) for 5 min. After differentiation with 1% hydrochloric acid alcohol for several seconds, the tissue sections were stained by eosin (Solarbio, #G1120) for 2 min, dehydrated by using gradient ethanol and sealed with neutral gum. Finally, the sections were observed under a microscope CKX53 (Olympus, Tokyo, Japan). Pulmonary vessel area and wall thickness were measured as previous described [36]: Pulmonary arterioles around 100 μm were selected for measurement, and 10 cross-sectional pulmonary arteries were randomly selected from each slice. Wall thickness (%) = (external diameter - internal diameter)/ external diameter×100%, and vessel area (%) = (total area - internal area) / total area×100%.

### Immunofluorescence staining

To measure the expression of NLRP3, PHD2 and HIF1α in pulmonary tissues, the paraffin-embedded lung tissue sections were prepared for immunofluorescence staining. The sections were firstly permeabilized with 0.1% Triton X-100 for 30 min, and blocked with 5% bovine serum albumin for 1 h. Then, the sections were incubated overnight with primary antibody against NLRP3 (Abcam, Cambridge, UK, #ab263899), PHD2 (Abcam, #ab226890) and HIF1α (Abcam, #ab179483) at 4 ℃, and incubated with Alexa Fluor®488 labeled IgG secondary antibody (Abcam, #ab150077). The nuclei were counterstained with Hoechst 33,258 (Abcam, #ab228550), and the image was visualized under a microscope and analyzed by the image J software. Density of NLRP3, PHD2 or HIF1α (%) = NLRP3 (PHD2 or HIF1α) positive cells / DAPI positive cells × 100%.

### Cell culture and treatment

Rat PASMCs (#YS1633C) were purchased from Yaji biological Co., Ltd (Shanghai, China), and cultured with DMEM medium containing 20% fetal bovine serum in an incubator supplemented with 5% CO_2_ at 37 ℃. Cells were cultured under normoxia condition (containing 21% O_2_) or hypoxia condition (containing 2% O_2_) for 24 h, and treated with or without AS-IV.

### Cell transfection

PASMCs were transfected with small interfering RNA (siRNA)-NC or siRNA-PHD2, and treated with 20 µM AS-IV, or treated with 10 µM HIF1α inhibitor LW6 (Solarbio, Beijing, China, #IL0860) alone. Cells were cultured in either normoxia condition or hypoxia condition for 24 h. siRNA-NC and siRNA-PHD2 were synthetized by Sangon Biotech (Shanghai, China), and they were transfected to PASMCs in the logarithmic phase using Lipofectamine 2000 transfection reagent (Invitrogen, California, USA, #11668030).

### Enzyme linked immunosorbent assay (ELISA)

Levels of IL-1β and IL-18 in rat serum or cell supernatant were measured by rat ELISA kit (Mlbio, Shanghai, China, #ml037361, #ml002816), and levels of matrix metallopeptidase (MMP2 or MMP9) and tissue inhibitors of metalloproteinase 4 (TIMP4) in pulmonary tissue homogenate or cell supernatant were detected by corresponding ELISA kit (Kanglang Biology, Shanghai, China, #KL12748Ra, #KL12754Ra, #KL12954Ra), according to the indicated manufacturer’s instructions.

### Western blot

Pulmonary artery tissue or PASMCs were homogenized in RIPA lysis buffer, and the protein concentration was quantified by BCA Protein Assay kit (Beyotime, Shanghai, China). Then, 30 µg total protein samples were separated 12% SDS-PAGE, transferred to a nitrocellulose membrane. The corresponding blots were cut prior to incubation with antibodies, blocked with 5% skim milk for 1 h at room temperature, and incubated with primary antibodies against NLRP3 (Abcam, #ab263899), cleaved Caspase-1 (Immunoway, Beijing, China, #YC0003), Fibronectin (Abcam, #ab268020), Collagen1 (Abcam, #ab260043), PHD2 (Abcam, #ab226890), HIF1α (Abcam, #ab179483), Gasdermin D N terminal fragment (GSDMD-N) (Abcam, #ab215203) and GAPDH (Abcam, #ab9484) at 4 ℃ overnight. The membranes were incubated with horseradish peroxidase-labeled IgG secondary antibody (Abcam, #ab6721) for 2 h. The blots were visualized with a gel imaging analysis system (Liuyi, Beijing, China, #WD-9413B), and the expression of all proteins was normalized to GAPDH.

### Masson staining

To evaluate lung fibrosis in PAH model rats, the pulmonary artery tissues were stained by using Masson staining reagent (Leagene biotechnology, Beijing, China, #DC0033), according to the manufacturer’s instruction. The stained sections were observed under a microscope: the collagen fiber was stained with blue, the cytoplasm was red, and the nucleus was blue brown.

### Propidium iodide (PI)/Hoechst double fluorescent staining

PASMCs to be detected were resuspended with cell staining buffer in 1.5 ml centrifuge tubes, and stained with 5 µL Hoechst 33,342 staining reagent (Solarbio, #CA1120) and 5 µL PI staining solution (Solarbio, #CA1120) at 4 ℃ for 30 min. The cells were centrifuged for precipitation, smeared and visualized under a microscope. The dead cells could be stained with red by PI solution, and PI-positive rate was analyzed by the ImageJ software.

### Lactate dehydrogenase (LDH) release assay

To evaluate pyroptotic cell death, LDH activity in PASMCs with corresponding treatment was detected with LDH Release Assay Kit (Beyotime, #C0016), according to manufacturer’s instruction. The absorbance at 490 nm was determined under a microplate reader (Thermo Fisher Scientific, Shanghai, China, #1410101), and the relative LDH activity was normalized to the control.

### Statistical analysis

Prism 8 software (GraphPad, California, USA) was used for statistical analysis. All data were presented as “mean ± standard deviation”, data among groups were compared by one-way ANOVA, and data between two groups were compared with LSD-t test. Difference with P values < 0.05 were considered statistically significant.

## Results

### AS-IV attenuates experimental PAH progression

**Fig. 1:**
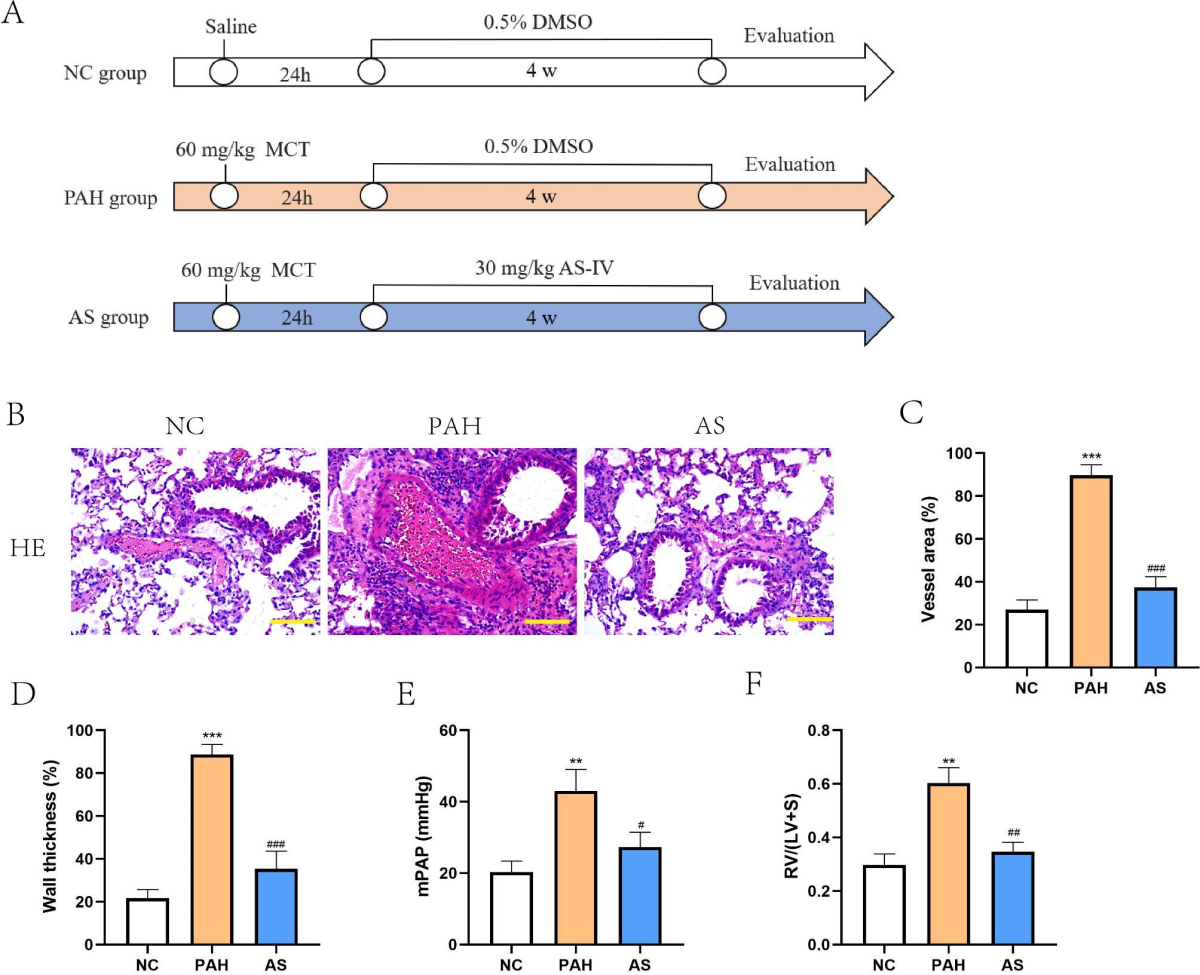
**AS-IV attenuated PAH progression in PAH model rats. A**, Schematic diagram displaying in vivo experiments procedures. **B**, Pathological changes of pulmonary artery in indicated rats was observed by hematoxylin-eosin staining. Scale bar = 100 μm. **C**-**D**, Pulmonary vessel area and wall thickness of pulmonary artery were measured. **E**, Analysis of mPAP in indicated rats. F, Weight ratio of the right ventricle to the left ventricle and the septum [RV/(LV + S)] was calculated. ^*^P<0.05, ^**^P<0.01, ^***^P<0.001, comparison with NC group. ^#^*P*<0.05, ^##^*P*<0.01, ^###^*P*<0.001, comparison with PAH group

To verify the effect of AS-IV on experimental PAH, the PAH rat models were established through administration of MCT, and then treated with AS-IV (Fig. 1A). By pathological staining of lung tissue, we observed that AS-IV improved pulmonary artery structural remodeling in PAH model rats, including vascular thickening or even lumen closure (Fig. 1B). Data in Fig. 1C and D also showed that increased vessel area and wall thickness in rat lung of PAH model rats were effectively reversed by AS-IV treatment. The PAH-associated elevation in mPAP and the index of right ventricle hypertrophy (RV/LV + S) were also observed in MCT-induced PAH model rats, and AS-IV significantly ameliorated these PAH-like changes (Fig. 1E and F). The results confirmed that AS-IV attenuates PAH progression in MCT-induced PAH model rats.

### AS-IV alleviates pyroptosis and fibrosis development in PAH model rats

**Fig. 2:**
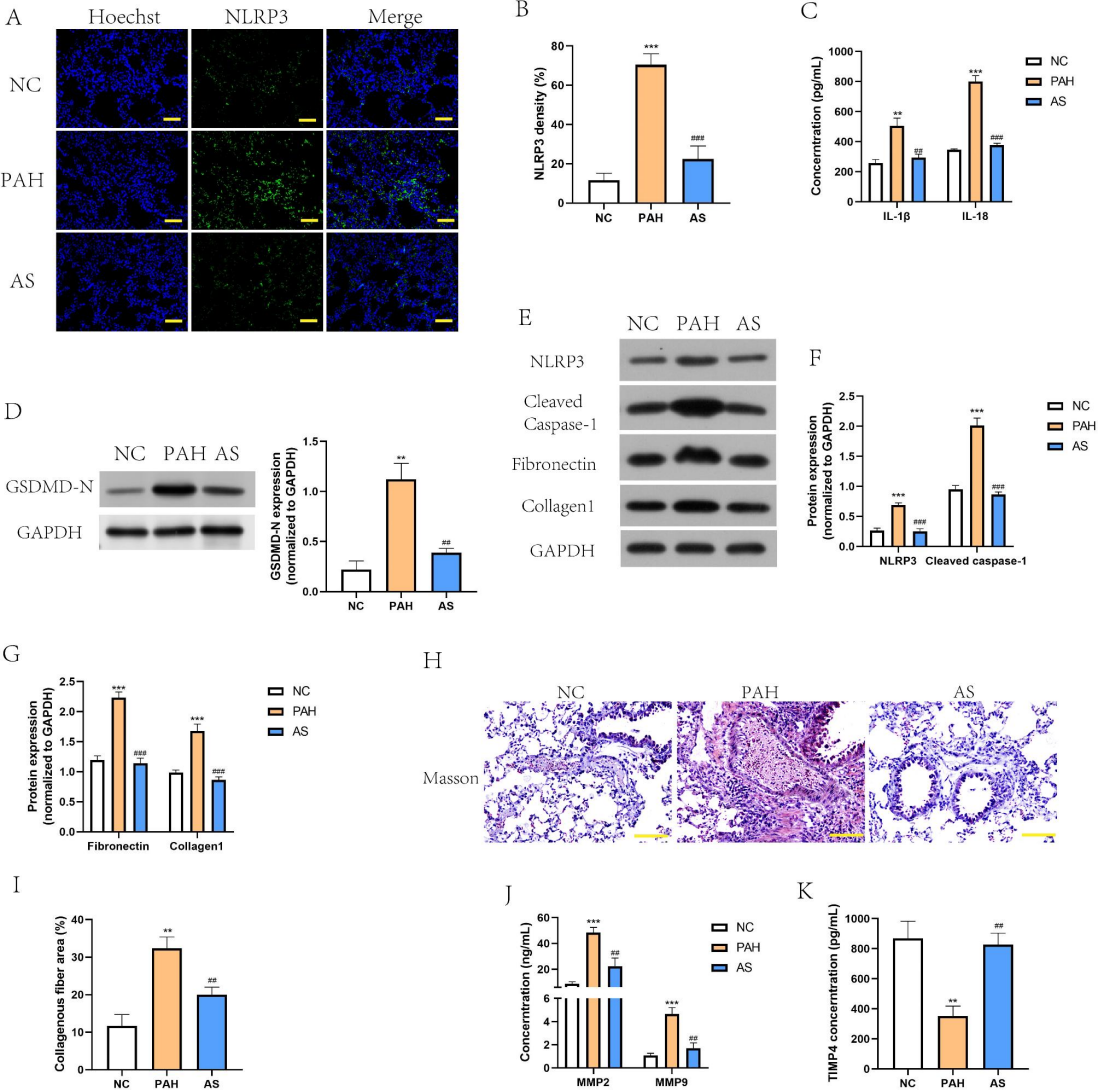
**AS-IV alleviated pyroptosis and fibrosis development in PAH model rats. A**, Immunofluorescence staining of NLRP3 in pulmonary tissue section of indicated rats. Scale bar = 50 μm. **B**, NLRP3 density in pulmonary tissues was quantified. **C**, IL-1β and IL-18 levels in rat serum were measured by ELISA. **D**-**G**, Cropped blots of GSDMD-N, NLRP3, cleaved Caspase-1, Fibronectin and Collagen1 and quantification of grayscale value (the samples derived from the same experiment and that gels/blots were processed in parallel). **H**-**I**, Degree of pulmonary fibrosis was evaluated by Masson staining. Scale bar = 100 μm. **J**-**K**, Proteins related to extracellular matrix metabolism, including MMP2/9 and TIMP4 were detected by ELISA method. ^**^P<0.01, ^***^P<0.001, comparison with NC group. ^##^*P*<0.01, ^###^*P*<0.001, comparison with PAH group

Pyroptosis and fibrosis are typical pathological characteristics that play critical role in PAH progression [24]. To evaluate the effect of AS-IV on pyroptosis phenomenon, we first conducted the immunohistochemical staining of NLRP3. As shown in Fig. 2A and B, NLRP3 expression was obviously increased in PAH model rats, but the change was mitigated by AS intervention. ELISA data indicated that AS-IV reduced the levels of inflammatory cytokines (IL-1β and IL-18) in pulmonary tissues PAH model rats (Fig. 2C). The subsequent western blot results also showed that elevated expression of GSDMD-N, NLRP3 and cleaved caspase-1 in lung tissues seen in PAH model rats was abrogated by As-IV administration (Fig. 2D, E and F), indicating that AS-IV alleviated pyroptosis in pulmonary artery tissues. To determine the change of fibrosis appearance after AS-IV intervention, the fibrosis markers were then detected by the western blot, and results demonstrated that AS-IV inhibited the production of fibronectin and collagen1 in PAH model rats (Fig. 2E and G). Additionally, Masson staining suggested that As-IV reduced collagen deposition and fibrosis development of pulmonary tissues in PAH model rats (Fig. 2H and I). Moreover, the proteins responsible for extracellular matrix metabolism, including MMP2/9 and TIMP4, were measured by ELISA method. Compared to rats in NC group, MMP2 and MMP9 were increased, but TIMP4 was decreased in pulmonary tissues of PAH model rats to promote extracellular matrix remodeling. However, the dysregulation of MMPs/TIMP4 was alleviated by the administration of As-IV (Fig. 2J and K). The findings indicated that AS-IV alleviates pyroptosis and fibrosis development in PAH model rats.

### AS-IV reduces pyroptosis and fibrosis development in hypoxia-treated PASMCs

**Fig. 3:**
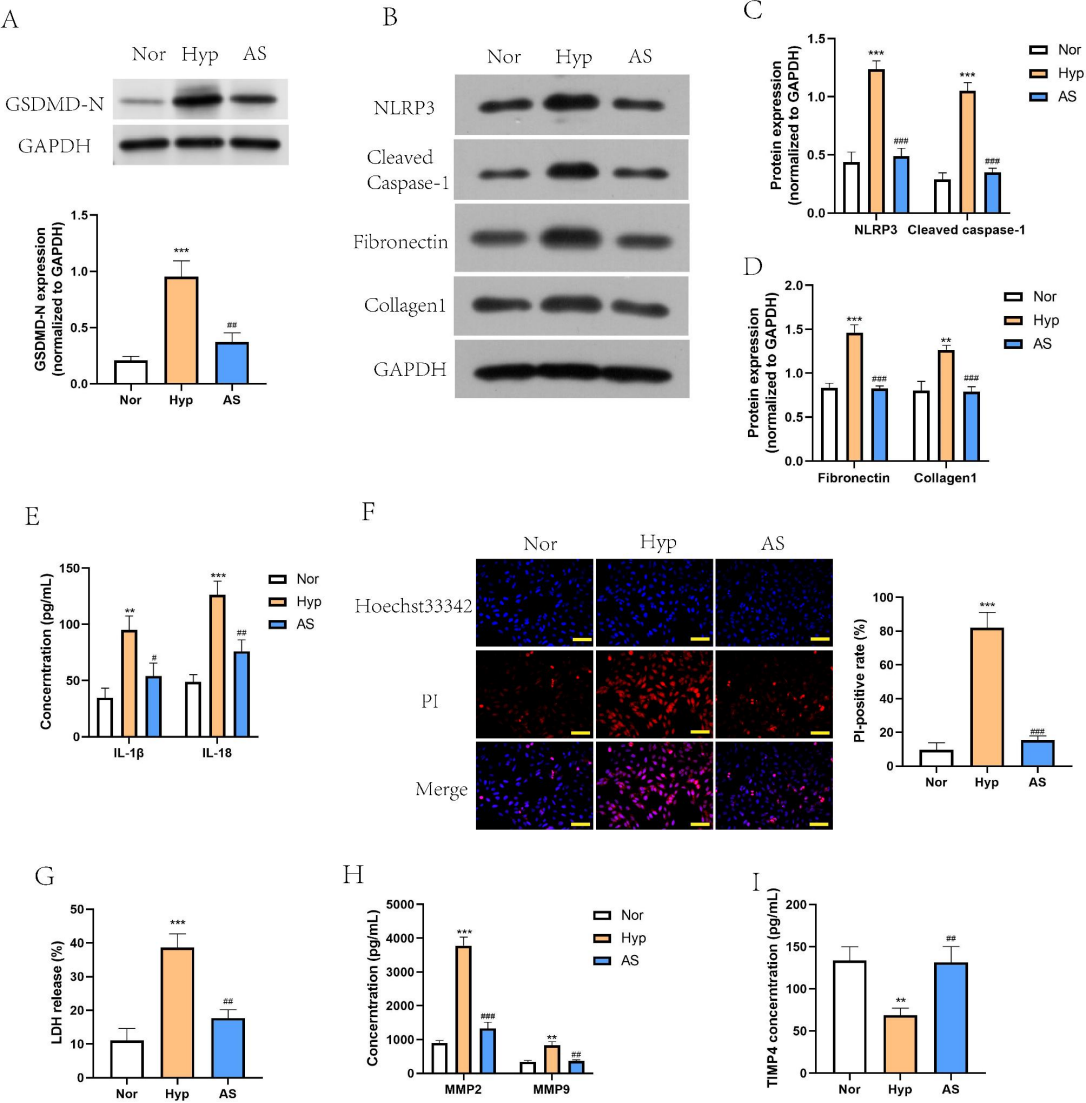
**AS-IV reduced pyroptosis and fibrosis development in hypoxia-treated PASMCs.** PASMCs were divided into 3 groups: Nor, Hyp and AS, cultured with normoxia or hypoxia, and treated with or without AS-IV. **A**-**D**, Cropped blots of GSDMD-N, NLRP3, cleaved Caspase-1, Fibronectin and Collagen1 and quantification of grayscale value (the samples derived from the same experiment and that gels/blots were processed in parallel). **E**, IL-1β and IL-18 levels in cell supernatant were measured by ELISA. **F**, Cell death was detected by PI/Hoechst double fluorescent staining. Scale bar = 50 μm. G, LDH activity was detected by a LDH release agent. **H**-**I**, Proteins related to extracellular matrix metabolism, including MMP2/9 and TIMP4 were detected by ELISA method. ^*^P<0.05, ^**^P<0.01, ^***^P<0.001, comparison with Nor group. ^#^*P*<0.05, ^##^*P*<0.01, ^###^*P*<0.001, comparison with Hyp group

Subsequently, we constructed cellular model of PAH with hypoxia-treated PASMCs. Similar to the *in vivo* models, hypoxia significantly promoted the expression of pyroptosis markers (GSDMD-N, NLRP3 and cleaved caspase-1) in PASMCs *in vitro*, which could be reversed by AS-IV (Fig. 3A C). Also, AS-IV abolished the elevation of IL-1β and IL-18 levels in hypoxia-treated PASMCs (Fig. 3E). Then, PI/Hoechst double fluorescent staining and LDH release assay were performed to evaluate pyroptosis phenomenon (Fig. 3F and G). Hypoxia increased the PI-positive rate and LDH activity in PASMCs, but AS-IV treatment decreased them, suggesting that AS-IV mitigated hypoxia-induced pyroptosis. In addition, we measured two fibrosis indexes (fibronectin and collagen1) by western blot (Fig. 3B and D), and data proved that AS-IV suppressed the production of fibrosis factors in hypoxia-treated PASMCs. Results from ELISA method showed that hypoxia treatment increased MMP2 and MMP9 levels, but decreased TIMP4 level in cell culture supernatant of PASMCs, which was also reversed by treatment of AS-IV (Fig. 3H and I). Consistent with the *in vivo* data, AS-IV reduces pyroptosis and fibrosis development in hypoxia-treated PASMCs *in vitro*.

### AS-IV affects the PHD2-HIF1α axis in PAH model rats and hypoxia-treated PASMCs

**Fig. 4:**
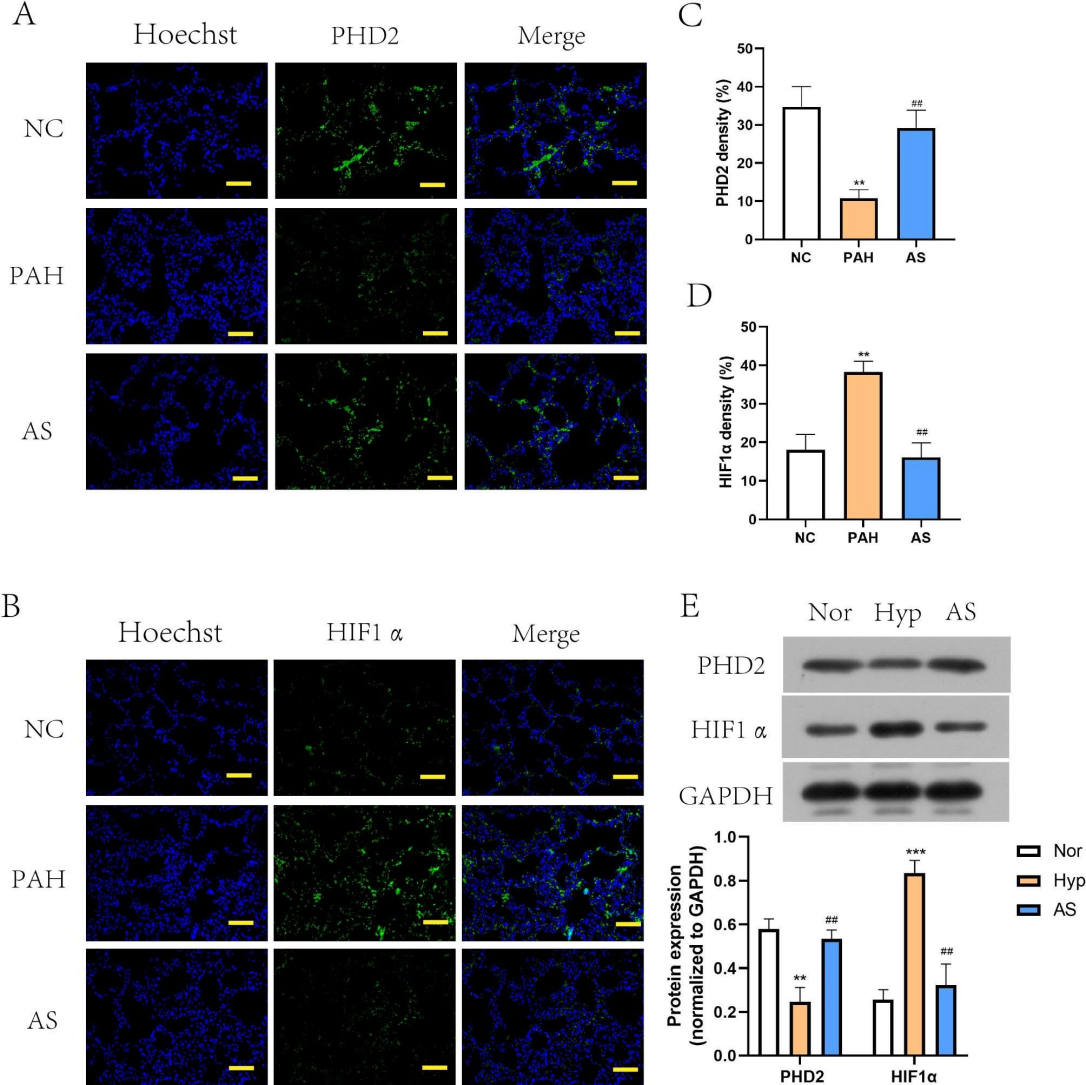
**AS-IV affected the PHD2-HIF1α axis in PAH model rats and hypoxia-treated PASMCs**. **A**-**B**, Immunofluorescence staining of PHD2 and HIF1α in pulmonary artery tissue section of the above rats. Scale bar = 50 μm. **C**-**D**, Quantification of PHD2 and HIF1α density in pulmonary tissues. ^***^P<0.001, comparison with NC group. ^###^*P*<0.001, comparison with PAH group. E, Cropped blots of PHD2 and HIF1α in above PASMCs and quantification of grayscale value (the samples derived from the same experiment and that gels/blots were processed in parallel). ^**^P<0.01, ^***^P<0.001, comparison with Nor group.^##^*P*<0.01, ^###^*P*<0.001, comparison with Hyp group

Since the suppressive effect of AS-IV on PAH development was observed *in vivo* and *in vitro*, the regulatory mechanism was then investigated. The PHD2/HIF1α axis is an important regulatory signal under hypoxia [37–39]. Through immunohistochemical staining of PHD2 and HIF1α in the pulmonary tissues of PAH model rats, we found that PHD2 expression was decreased in pulmonary tissues of PAH model rats, while HIF1α expression was markedly elevated, which was reversed by AS-IV administration (Fig. 4A, B, C and D). Consistent with the *in vivo *results, data from western blot assay also proved that AS-IV promoted PHD2 expressions and suppressed HIF1α production in hypoxic PASMCs (Fig. 4E). The results illustrated that AS-IV affects the PHD2-HIF1α axis *in vivo* and *in vitro*.

### AS-IV depresses pyroptosis and fibrosis development in hypoxia-treated PASMCs through the PHD2-HIF1α axis

**Fig. 5:**
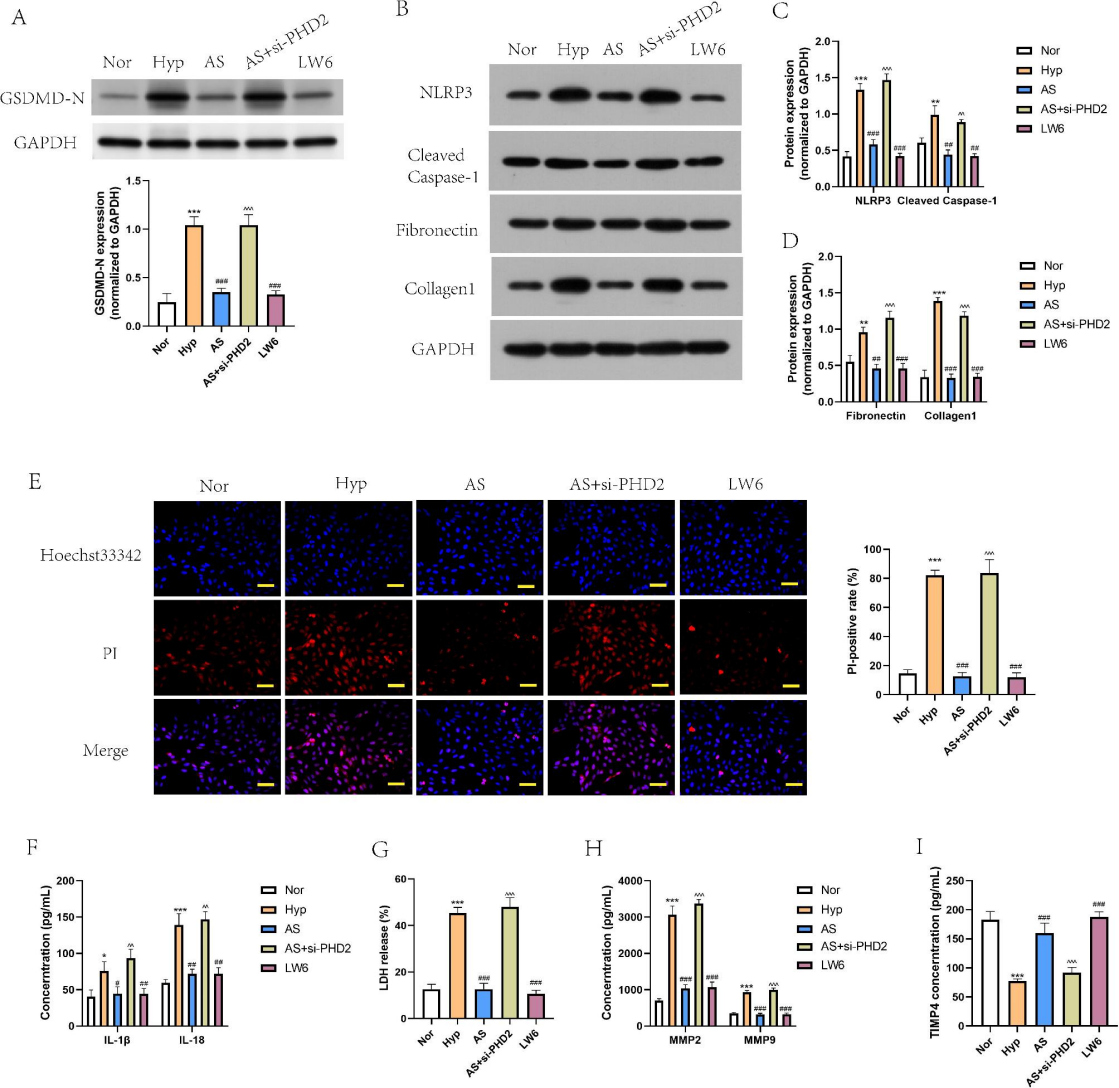
**AS-IV depressed pyroptosis and the production of fibrosis markers induced by hypoxia in PASMCs through the PHD2-HIF1α axis. A**-**D**, PASMCs were divided into 5 groups: Nor, Hyp, AS, AS + si-PHD2 and LW6. Cropped blots of GSDMD-N, NLRP3, cleaved Caspase-1, Fibronectin and Collagen1 (the samples derived from the same experiment and that gels/blots were processed in parallel), and quantitation of the pyroptosis markers and the fibrosis markers. **E**, Cell death was detected by PI/Hoechst double fluorescent staining. Scale bar = 50 μm. **F**, IL-1β and IL-18 levels in cell supernatant were measured by ELISA. G, LDH activity was detected by a LDH release agent. **H**-**I**, Proteins of MMP2/9 and TIMP4 were detected by ELISA method. ^*^P<0.05, ^**^P<0.01, ^***^P<0.001, comparison with Nor group. ^#^*P*<0.05, ^##^*P*<0.01, ^###^*P*<0.001, comparison with Hyp group.^^^*P*<0.05, ^^^^*P*<0.01, ^^^^^*P*<0.001, comparison with AS group

To explore whether and how PHD2-HIF1α axis involved in the regulation of AS-IV on PAH, the function verification experiments were performed in hypoxia-treated PASMCs. Hypoxic PASMCs were pre-transfected with siRNA-PHD2 and administered with AS-IV, or treated with LW6, a HIF1α inhibitor. Then, we found that both AS-IV and LW6 decreased the levels of pyroptosis markers in hypoxia-treated PASMCs, including GSDMD-N, NLRP3 and cleaved caspase-1 (Fig. 5A-C), and suppressed cell death (Fig. 5E), release of IL-1β and IL-18 (Fig. 5F), and LDH activity (Fig. 5G), but co-treatment with siRNA-PHD2 and AS-IV abolished the suppressive effect on pyroptosis of AS-IV. In addition, the expression of fibronectin and collagen1 in hypoxia-treated PASMCs were decreased by both AS-IV and LW6, and the suppressive effect of AS-IV on fibrosis factors was also reversed by PHD2 knockdown (Fig. 5B and D). Furthermore, it was found that inhibiting HIF1α exerted similar effect to AS-IV on hypoxia-induced MMPs/TIMP4 dysregulation, while downregulation of PHD2 restored the levels of MMP2 and MMP9, but suppressed TIMP4 level, counteracting the regulatory effect of AS-IV on hypoxia-triggered MMPs/TIMP4 imbalance in PBMCs (Fig. 5H and I). Taken together, the above data suggested that AS-IV depresses hypoxia-induced pyroptosis and fibrosis development in PASMCs through regulating the PHD2-HIF1α axis.

## Discussion

To date, PAH is still an incurable chronic disease characterized by the progressive increase of pulmonary artery pressure caused by pulmonary vascular inflammation and pulmonary vascular remodeling [40]. Searching for safe, effective and low-cost specific drugs has become an urgent challenge in the research field. AS-IV, as the main active components of *Astragalus membranaceus*, has a good anti-inflammatory effect in diabetes, ischemia-reperfusion injury, allergic diseases, etc. [41–43]. In the present study, by using MCT-induced PAH model rats and hypoxia-treated PASMCs, we verified that AS-IV alleviated MCT-induced pulmonary artery structural remodeling, pulmonary hypertension and right ventricular hypertrophy, and suppressed NLRP3-mediated pyroptosis and fibrosis in PAH models *in vivo* and *in vitro*. Pyroptosis, also known as inflammatory necrosis, is a kind of programmed cell death triggered by NLRP3 inflammasome activation [44, 45]. It is manifested as sequential cleavage and activation of caspase-1 and GSDMD and the rupture of cell membrane, resulting in the release of proinflammatory cytokines (such as IL-1β and IL-18) and the activation of strong inflammatory response [46]. NLRP3-mediated pyroptosis in PASMCs is found to be involved in the occurrence and development of atherosclerosis and pulmonary hypertension [47, 48]. We observed that AS-IV inhibited the expression of NLRP3, cleaved caspase-1, GSDMD-N, the release of IL-1β and IL-18 in PAH model rats or hypoxia-induced PASMCs, indicating the suppressive effect of AS-IV on pyroptosis during PAH. In addition, pyroptosis has been proved to a trigger of fibrosis, and parenchymal cells regenerate after pyroptosis to replace necrotic cells during injury, which is considered to be a result of wound healing response to repeated injury [49, 50]. In our study, MCT or hypoxia increased fibrosis markers (fibronectin and collagen1) and MCT-induced collagen deposition in pulmonary tissue were suppressed by AS-IV treatment. Pyroptosis is a special type of programmed cell death that can quickly activate inflammatory reactions [51]. Most of the current publications focused on the over-proliferation of PASMCs, and little attention has been paid to the pyroptosis of PASMCs, and in fact, these two situations co-exist in PASMCs of PAH models [11, 52]. The intense inflammatory response caused by pyroptosis can lead to an imbalance in the ratio of MMPs and TIMPs (which were responsible for ECM metabolism), leading to extracellular matrix remodeling, collagen deposition, and fibrosis development [3]. In our study, AS-IV reversed the increase of MMP2 and MMP9 and the decrease of TIMP4 in pulmonary tissues of PAH model rats and cell culture supernatant of hypoxic PASMCs.

Herein, we identified a novel signaling pathway, the PHD2/HIF1α axis, as the main regulatory mechanism of AS-IV in PAH. HIF1α, recognized as a cellular oxygen receptor, is a key regulatory signal in cells under hypoxia [53]. Under normoxia condition, HIF1α is instability due to the hydroxylation of proline by PHD proteins containing hydroxylase domains and the subsequent ubiquitination degradation [54]. During hypoxia, the deficiency of oxygen leads to a reduction of the hydroxylation effect of prolyl hydroxylase proteins on HIF1α, and non-hydroxylated HIF1α is more stable than hydroxylated HIF1α, resulting in increased protein expression [55, 56]. HIF1α can transfer to the nucleus, and regulate the expression of related target genes, including NLRP3 [57–59]. Therefore, identifying naturally occurring HIF1α inhibitors can provide new insights into the development of PAH drugs. Previous evidences found that PHD2 deficiency contributes to vascular remodeling, hypertension and right ventricular hypertrophy during experimental PAH development [31, 39, 60]. In this study, we found that AS-IV elevated PHD2 to suppress HIF1α, thus suppressing MCT or hypoxia-induced pyroptosis and fibrosis, and restraining PAH development. A recent study by Sun Y [35] points out that AS-IV inhibits MCT-induced inflammatory response through NLRP3/calpain-1 pathway, which partially supports our results. Another study by Yao J [9] suggests that AS-IV alleviates hypoxia-induced pulmonary vascular remodeling by modulating Notch signaling pathway. Differently, our research not only investigated inflammation, but also proved the effect of AS-IV on pyroptotic necrosis and fibrosis, and revealed the upstream molecular mechanism, providing a novel mechanism for the pulmonary fibrogenesis and PAH progression.

## Conclusion

**Fig. 6:**
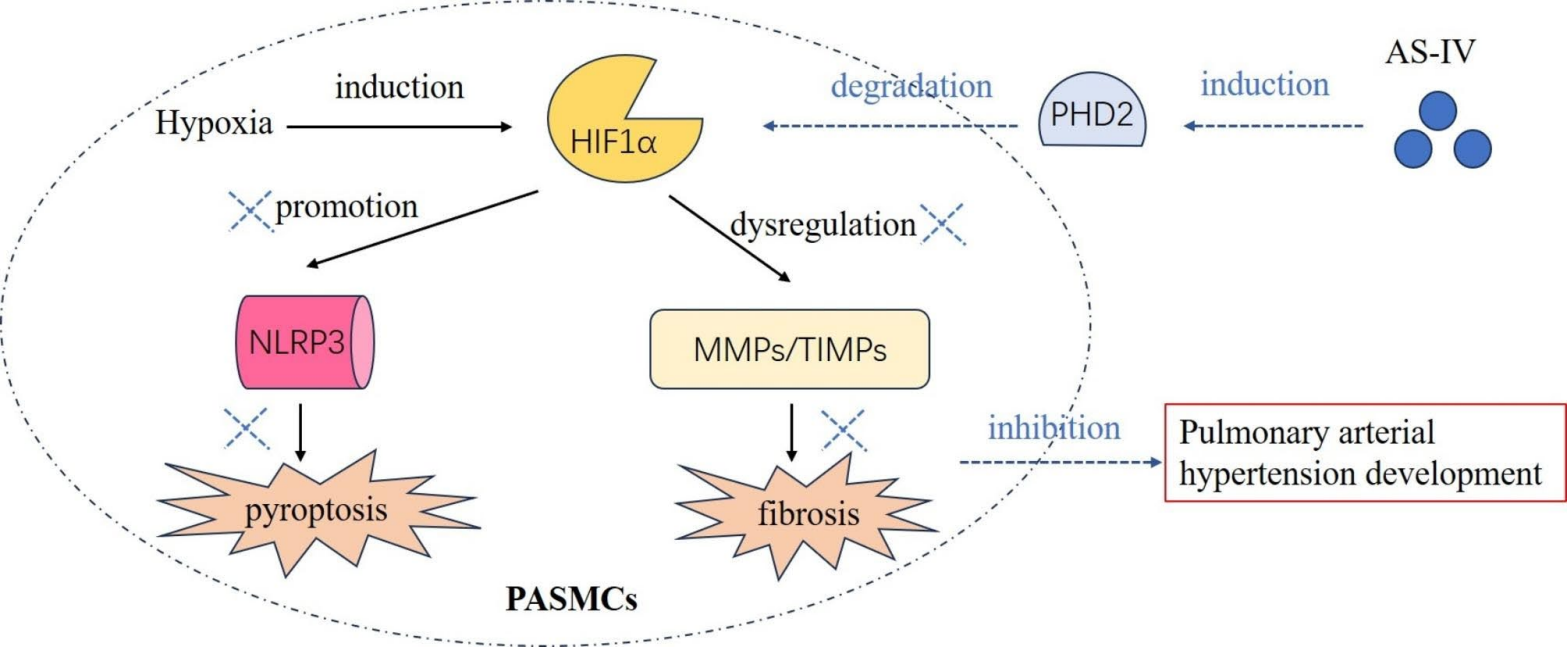
**Mechanism’s diagram.** AS-IV elevates PHD2 expression to accelerate HIF1α degradation, and then suppresses NLRP3-mediated pyroptosis and MMPs/TIMPs-mediated fibrosis development in hypoxia-induced PASMCs, thus restraining PAH progression

In summary, we verified that AS-IV can alleviate pulmonary artery structural remodeling and pulmonary hypertension development in experimental PAH models. Mechanistically, we uncovered for the first time that AS-IV suppresses NLRP3-mediated pyroptosis and fibrosis in PASMCs through modulating the PHD2/HIF1α signaling pathway, thus restraining PAH progression (Fig. 6). The findings reveal the potential mechanisms of AS-IV in PAH treatment, and illustrate the PHD2/HIF1α may be a potential anti-pyroptosis target during PAH. Greater efforts will be needed in the future to validate the clinical efficiency of AS-IV in the management of PAH.

### Electronic supplementary material

Below is the link to the electronic supplementary material.

Supplementary Material 1

Supplementary Material 2

## Data Availability

The data were all included in this paper. The original data are available from the corresponding author upon reasonable request.

## Abbreviations

PAH: pulmonary artery hypertension
AS: Astragaloside
MCT: monocrotaline
mPAP: mean pulmonary artery pressure
RV/(LV + S): weight ratio of the right ventricle to the left ventricle plus the septum
IL-1β: interleukin-1β
IL-18: interleukin-18
MMP2/9: matrix metallopeptidase 2\9
TIMP4: tissue inhibitors of metalloproteinase 4
LDH: lactate dehydrogenase
NLRP3: nucleotide-binding oligomerization segment-like receptor family 3
PASMCs: pulmonary arterial smooth muscle cells
PI: propidium iodide
qRT-PCR: quantitative reverse-transcription PCR
siRNA: small-interfering RNA
PHD2: prolyl-4-hydroxylase 2
HIF1α: hypoxia inducible factor-1α
GSDMD-N: Gasdermin D N terminal fragment
