# Supplementary material for: Astragaloside IV restrains pyroptosis and fibrotic development of pulmonary artery smooth muscle cells to ameliorate pulmonary artery hypertension through the PHD2/HIF1α signaling pathway
Source: BMC Pulm Med. 2023 Oct 12;23:386. doi: 10.1186/s12890-023-02660-9 (PMC10568875; doi:10.1186/s12890-023-02660-9)
Supplement: Supplementary file 1 — Supplementary Material 1 [file 12890_2023_2660_MOESM1_ESM.docx]

**Comparison of cropped blots and original blots**


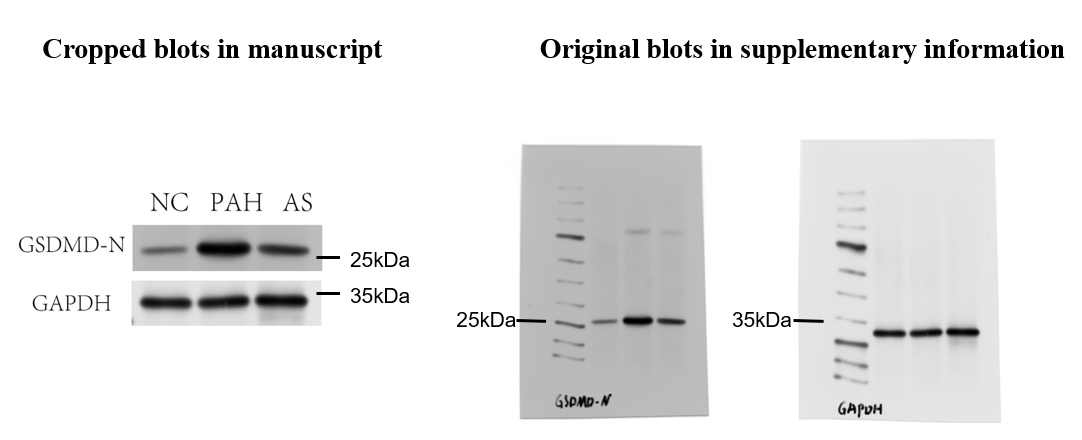


**Figure 2D**


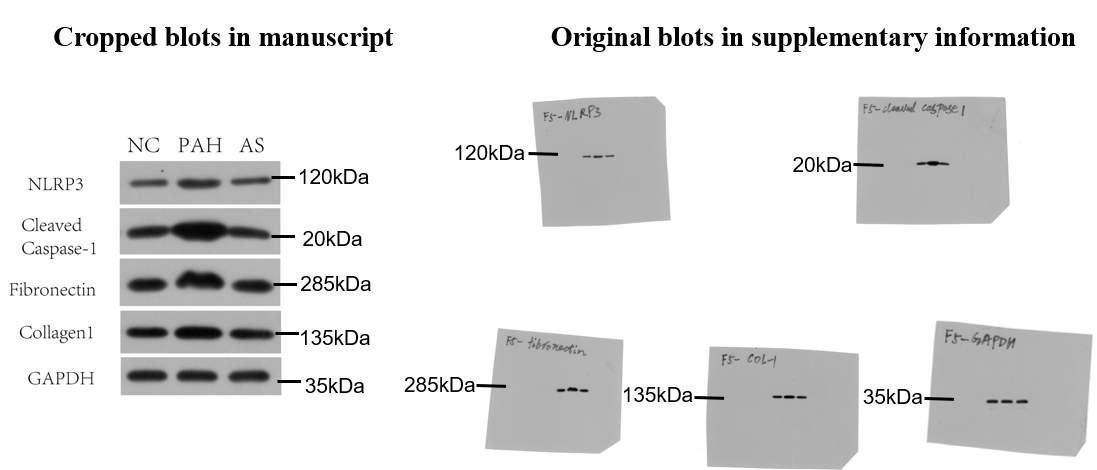


**Figure 2 E**


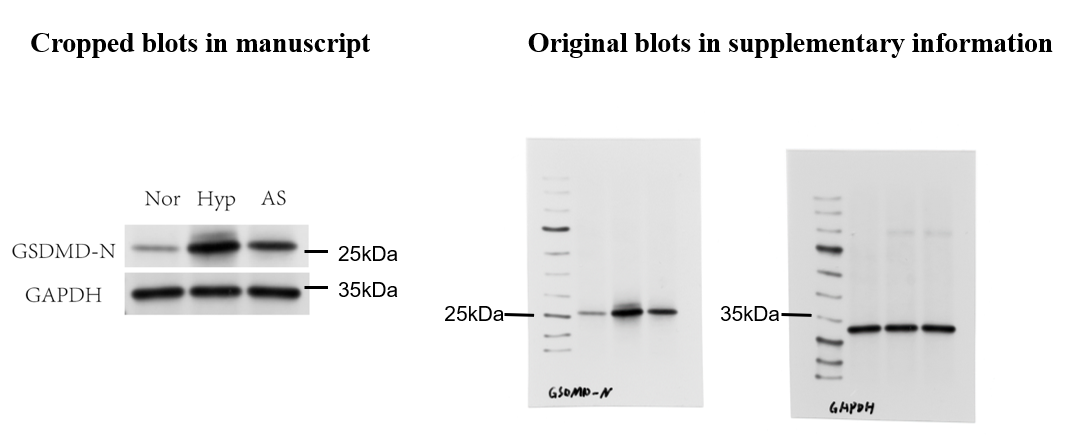


**Figure 3A GSDMD-N and GAPDH**


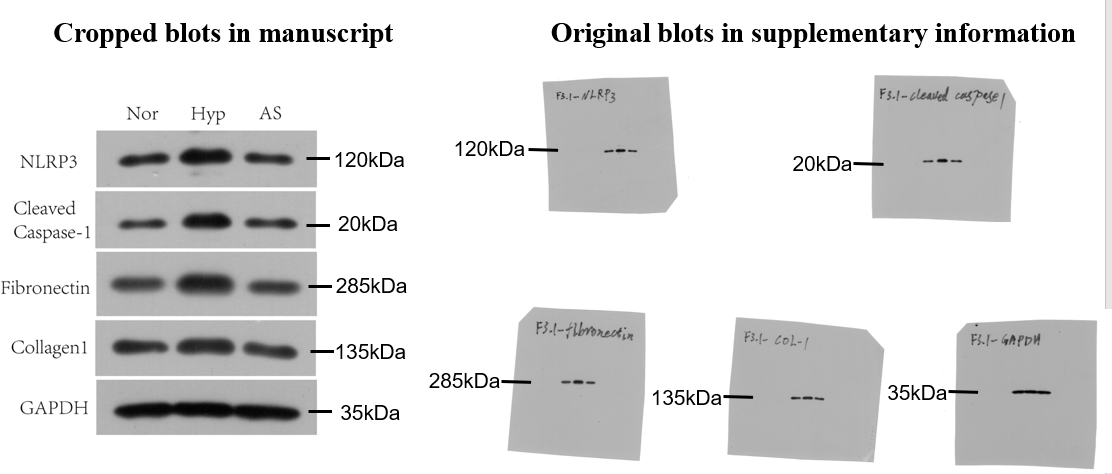


**Figure 3 B**


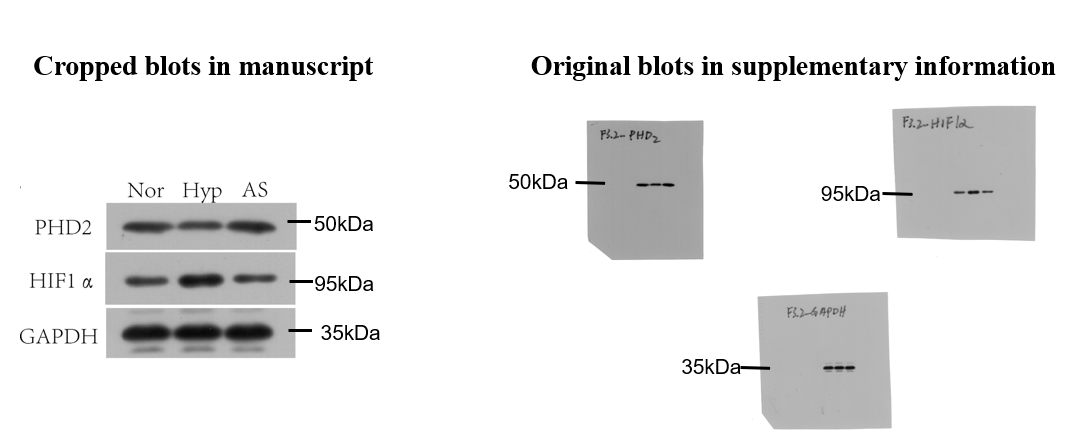


**Figure 4 E**


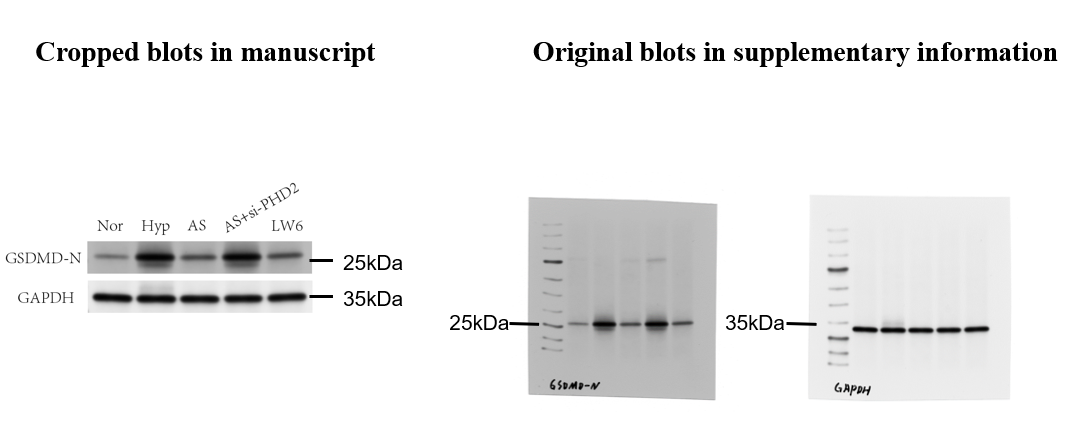


**Figure 5A GSDMD-N and GAPDH**


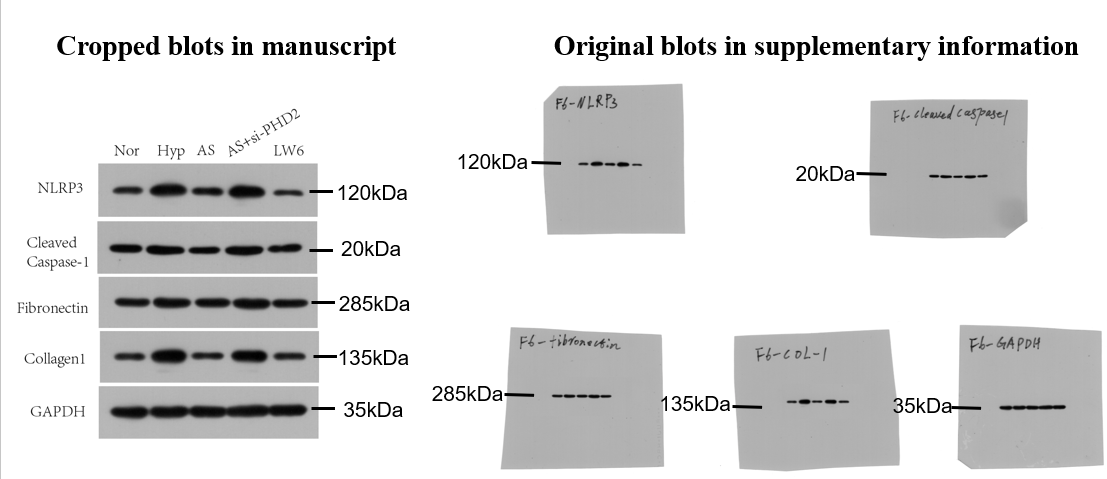


**Figure 5B**
