# Supplementary material for: Astragaloside IV restrains pyroptosis and fibrotic development of pulmonary artery smooth muscle cells to ameliorate pulmonary artery hypertension through the PHD2/HIF1α signaling pathway
Source: BMC Pulm Med. 2023 Oct 12;23:386. doi: 10.1186/s12890-023-02660-9 (PMC10568875; doi:10.1186/s12890-023-02660-9)
Supplement: Supplementary file 2 — Supplementary Material 2 [file 12890_2023_2660_MOESM2_ESM.docx]

**Supplemented western blots images**

**Figure 2D GSDMD-N**

**Figure 2D GAPDH**

**Figure 2E NLRP3**

**Figure 2E cleaved caspase-1**

**Figure 2E Fibronectin**

**Figure 2E Collagen1**

**Figure 2E GAPDH**

**Figure 3A GSDMD-N**

**Figure 3A GAPDH**

**Figure 3B NLRP3**

**Figure 3B cleaved caspase-1**

**Figure 3B Fibronectin**

**Figure 3B Collagen1**

**Figure 3B GAPDH**

**Figure 4E PHD2**

**Figure 4E HIF1α**

**Figure 4E GAPDH**

**Figure 5A GSDMD-N**

**Figure 5A GAPDH**

**Figure 5B NLRP3**

**Figure 5B cleaved caspase-1**

**Figure 5B Fibronectin**

**Figure 5B Collagen1**

**Figure 5B GAPDH**
